# Characterizing the DNA Methyltransferases of *Haloferax volcanii* via Bioinformatics, Gene Deletion, and SMRT Sequencing

**DOI:** 10.3390/genes9030129

**Published:** 2018-02-27

**Authors:** Matthew Ouellette, J. Peter Gogarten, Jessica Lajoie, Andrea M. Makkay, R. Thane Papke

**Affiliations:** Department of Molecular and Cell Biology, University of Connecticut, Storrs, CT 06268, USA; matthew.ouellette@uconn.edu (M.O.); gogarten@uconn.edu (J.P.G.); jessica.lajoie@uconn.edu (J.L.); andrea.makkay@uconn.edu (A.M.M.)

**Keywords:** haloarchaea, *Halobacteria*, *Haloferax volcanii*, DNA methylation, methylation, methylome, restriction-modification system, CTAG methylation, GATC methylation

## Abstract

DNA methyltransferases (MTases), which catalyze the methylation of adenine and cytosine bases in DNA, can occur in bacteria and archaea alongside cognate restriction endonucleases (REases) in restriction-modification (RM) systems or independently as orphan MTases. Although DNA methylation and MTases have been well-characterized in bacteria, research into archaeal MTases has been limited. A previous study examined the genomic DNA methylation patterns (methylome) of the halophilic archaeon *Haloferax volcanii*, a model archaeal system which can be easily manipulated in laboratory settings, via single-molecule real-time (SMRT) sequencing and deletion of a putative MTase gene (*HVO_A0006*). In this follow-up study, we deleted other putative MTase genes in *H. volcanii* and sequenced the methylomes of the resulting deletion mutants via SMRT sequencing to characterize the genes responsible for DNA methylation. The results indicate that deletion of putative RM genes *HVO_0794*, *HVO_A0006*, and *HVO_A0237* in a single strain abolished methylation of the sole cytosine motif in the genome (C^m4^TAG). Amino acid alignments demonstrated that *HVO_0794* shares homology with characterized cytosine CTAG MTases in other organisms, indicating that this MTase is responsible for C^m4^TAG methylation in *H. volcanii*. The CTAG motif has high density at only one of the origins of replication, and there is no relative increase in CTAG motif frequency in the genome of *H. volcanii*, indicating that CTAG methylation might not have effectively taken over the role of regulating DNA replication and mismatch repair in the organism as previously predicted. Deletion of the putative Type I RM operon *rmeRMS* (*HVO_2269-2271*) resulted in abolished methylation of the adenine motif in the genome (GCA^m6^BN_6_VTGC). Alignments of the MTase (HVO_2270) and site specificity subunit (HVO_2271) demonstrate homology with other characterized Type I MTases and site specificity subunits, indicating that the *rmeRMS* operon is responsible for adenine methylation in *H. volcanii*. Together with HVO_0794, these genes appear to be responsible for all detected methylation in *H. volcanii*, even though other putative MTases (*HVO_C0040*, *HVO_A0079*) share homology with characterized MTases in other organisms. We also report the construction of a multi-RM deletion mutant (*ΔRM*), with multiple RM genes deleted and with no methylation detected via SMRT sequencing, which we anticipate will be useful for future studies on DNA methylation in *H. volcanii*.

## 1. Introduction

In bacteria and archaea, DNA methylation by DNA methyltransferases (MTases) has many roles. MTases are commonly associated with restriction-modification (RM) systems, in which the MTase functions alongside a cognate restriction endonuclease (REase). The REase will target the same sites of DNA as the MTase and cleave those that are unmethylated, whereas methylated motifs will be disregarded. RM systems function in self-recognition, allowing the host to differentiate between its own methylated DNA and potentially harmful foreign unmethylated DNA, which can then be recognized and digested by the REase [1,2]. RM systems have also been characterized as Toxin/Antitoxin systems that lead to addiction through post-segregational killing in case the methylation activity decays in a cell from which the RM system has been lost so that unmethylated restriction sites become exposed to the remaining restriction enzyme activity [3,4]. 

DNA methylation occurs at adenine or cytosine bases, resulting in one of three possible types of methylation: *N*6-methyladenine (6mA), *N*4-methylcytosine (4mC), or *C*5-methylcytosine (5mC) [5]. Methylation is catalyzed by DNA MTases, which interact with the cofactor *S*-adenosyl methionine (AdoMet) to transfer a methyl group to a nucleotide base of a DNA molecule. MTases typically consist of three major domains: an AdoMet binding domain which interacts with AdoMet to obtain the methyl group, a target recognition domain (TRD) which recognizes a short sequence of DNA to be targeted for methylation, and a catalytic domain which transfers the methyl group from AdoMet to the targeted nucleotide [6]. MTases can be categorized based on the type of methylation they perform (6mA, 4mC, and 5mC), with the 6mA and 4mC MTases being more similar to each other than to 5mC MTases [7,8]. MTases have been further categorized into subtypes based on the order of several conserved motifs that make up the primary domains of the MTases. The 6mA and 4mC MTases can be classified into six categories (α, β, γ, δ, ε, and ζ) based on the N-terminal to C-terminal order of conserved motifs X, I–III (AdoMet binding motifs), IV-VIII (catalytic motifs), and the TRD [8,9]. The occurrence of signature AdoMet binding motif DPPY and catalytic motif FXGXG (abbreviated FGG) can also be used to categorize these MTases [10]. The 5mC MTases have a different set of motifs that can be used to identify them [11,12].

There are four major types of characterized RM systems [13,14]. Type I RM systems consist of pentamer complexes with two REase (R) subunits, two MTase (M) subunits, and one site specificity (S) subunit containing two tandem TRDs which recognize bipartite target sites. When the complex comes across a target site, it will either methylate the site if it is methylated on one strand (hemimethylated) or will cleave the DNA several bases upstream or downstream from the site if it is unmethylated on both strands [15,16]. Type II RM systems include MTases and REases which operate independently and target the same sites of DNA [17]. Many subgroups of Type II RM systems have been categorized, such as Type IIG which consists of independent RM enzymes capable of both MTase and REase activity [18]. In Type III RM systems, a REase subunit (Res) and MTase subunit (Mod) work together in a two-component complex, with the Mod subunit containing the TRD which recognizes the target site of the system [19]. Type IV RM systems consist only of REases, and these REases cleave methylated target sites instead of unmethylated sites [20].

MTases can also occur independently in bacteria and archaea without cognate REases. These MTases, known as orphan MTases, typically provide important functions for their host organisms [21]. In *Escherichia coli*, for example, the orphan adenine MTase Dam is involved in coordinating timing of DNA replication by methylating GATC sites at the origin of replication which are also targets of binding for SeqA in a hemimethylated state [22]. When SeqA binds to hemimethylated GATC sites of the origin after replication has occurred, DnaA is prevented from binding to the origin and re-initiating DNA replication [23,24]. Dam methylation is also important in the methyl-directed mismatch repair (MMR) system in *E. coli*, in which the complex binds to a closely located methylated GATC site on the old strand in order to target and cleave the mismatched base on the new strand [25,26,27]. In *Caulobacter crescentus*, the orphan adenine MTase CcrM is involved in regulating the expression of genes like *ctrA*, which are essential for cell cycle regulation [28,29]. Orphan MTases can also protect the host from parasitic RM systems by mimicking the methylation of the invader, such as orphan cytosine MTase Dcm in *E. coli* which methylates the same sites as RM system EcoRII and prevents degradation of the genome by the invading system [30]. Orphan MTases are more common among the bacteria and more well-conserved within a genus than RM-associated MTases, likely due to orphan MTases performing important roles within their hosts [31,32].

DNA methylation has been well-studied in bacterial organisms. However, research has not been as extensive in the archaea, which have focused on characterizing methylation and a few RM systems primarily in thermophilic organisms [33,34,35,36]. A previous study [37] examined the genomic methylation patterns (methylome) of the halophilic archaeal organism *Haloferax volcanii*, a member of Class Halobacteria which are often referred to as haloarchaea, as a model for examining DNA methylation and RM systems in archaea, due to its well-established genetic system which allows it to be easily manipulated in lab settings [38,39]. In the study, the methylome of *H. volcanii* was sequenced via single-molecule real-time (SMRT) sequencing developed by Pacific Biosciences (PacBio) [40]. *H. volcanii* was observed to have two types of motifs methylated throughout its genome: C^m4^TAG and GCA^m6^BN_6_VTGC. The study also demonstrated that deletion of one of the putative RM genes (*HVO_A0006*) resulted in an alteration in the adenine motif, which was surprising considering that the gene is not a MTase but instead encodes an REase family protein [37]. In this follow-up study, we aim to characterize the MTases of *H. volcanii* through bioinformatics and gene deletions of the various predicted RM genes in the genome and sequence the methylomes of the deletion mutants via SMRT sequencing. We will also describe the production of an RM null mutant without a methylated genome, which we anticipate will be useful in future research of DNA methylation in the archaea.

## 2. Materials and Methods 

### 2.1. Strains and Growth Conditions

All strains and plasmids used in this study are listed and described in Table 1. Strains of *H. volcanii* were grown at 42 °C while shaking at 200 rpm using either rich medium (Hv-YPC) or selective rich medium (Hv-Ca) developed by Allers et al. [41] and outlined in the Halohandbook [42]. For ∆*pyrE2* strains, media was supplemented with uracil (50 µg/mL) and 5-fluoroorotic acid (50 µg/mL) as needed. Strains of *E. coli* were grown at 37 °C while shaking at 200 rpm in either Lysogeny Broth (LB) or S.O.C. medium (Clontech, Mountain View, CA, USA). Ampicillin (100 µg/mL) and X-gal (20 µg/mL) were added to the media when needed.

### 2.2. Deletion of Annotated Restriction Modification Genes

Putative RM genes in *H. volcanii* were identified from New England BioLabs Restriction Enzyme Database (REBASE) [10] and National Center for Biotechnology Information (NCBI) (Table 2). These genes were deleted in *H. volcanii* strain H1206 utilizing a method developed by Blaby et al. [39] that uses the In-Fusion HD Cloning Kit (Clontech). Primers were designed to construct deletion plasmids of putative RM genes and are listed in Table 3. These deletion plasmids were then used to transform *H. volcanii* H1206 and its derivatives via the polyethylene glycol (PEG)-mediated transformation protocol outlined in the Halohandbook [42]. Transformed cell cultures were plated on Hv-Ca and incubated at 42 °C for 5–7 days. Pop-ins were detected via a colony PCR screen using external deletion primers and visualized via gel electrophoresis. Confirmed pop-ins were then plated on Hv-Ca with 50 μg/mL 5-fluoroorotic acid (5-FOA) and 50 μg/mL uracil to pop-out genes of interest. Successful pop-outs were identified via PCR screen as performed for detecting pop-ins. Final deletion strains obtained though this method are listed in Table 1.

### 2.3. DNA Purification for Single-Molecule Real-Time Sequencing

In order to extract DNA from the *H. volcanii* deletion mutants for SMRT sequencing, 40 mL of cell cultures in late log to early stationary phase (optical density (OD_600_) = ~0.8–1) were pelleted and lysed by resuspension in 10 mM Tris-HCl buffer (pH 8.0). The lysates were then treated with proteinase K (50 μg/mL final concentration) and incubated overnight at 37 °C to hydrolyze the proteins, after which the DNA was extracted via ethanol precipitation. Performing three rounds of phenol-chloroform and two rounds of chloroform extractions purified the DNA further. A final ethanol precipitation was then performed on the remaining DNA, and the samples were purified of RNA via Agencourt AMPure XP beads (Beckman Coulter, Brea, CA, USA). The 260/280 ratio, 260/230 ratio, and DNA concentration of each sample was quantified via Nanodrop and Qubit dsDNA BR assay (Invitrogen, Eugene, OR, USA).

### 2.4. Single-Molecule Real-Time Sequencing

The DNA samples extracted from the *H. volcanii* deletion mutants were analyzed via PacBio SMRT sequencing in order to determine the methylomes of the strains. The samples were submitted to the Keck Sequencing Facility of the Yale School of Medicine for SMRT sequencing analysis. A detailed outline of the SMRT sequencing strategy can be found in the PacBio manual “Detecting DNA Base Modifications: SMRT Analysis of Microbial Methylomes” [46]. Libraries of 0.25 to 3 kb were constructed for each strain using an estimated input size of 4 Mb, and were each sequenced in one SMRT cell, resulting in coverage of ~150x for *∆RM*, ~400x for ∆*HVO_0794* ∆*HVO_A0006* ∆*HVO_A0237*, and ~120x for ∆*rmeRMS*. The SMRT Portal program “RS_Modification_and_Motif_Analysis.1” was used under default settings to determine the modified bases and motifs in ∆RM. The modified bases and motifs in the other strains were identified using the same SMRT Portal program, but with the ∆RM analysis results used as a control. All analyses used the *H. volcanii* DS2 genome as the reference sequence [47].

### 2.5. Bioinformatics Analysis

Homologs of the putative RM proteins in *H. volcanii* DS2 were discovered via protein BLAST (blastp) [48] and position-specific iterative BLAST (PSI-BLAST) of the non-redundant protein database on NCBI as well as translated nucleotide BLAST (tblastn) of the NCBI Halobacteria genome database (taxid 183963) (*E*-value cutoff of 1e^−4^). Homologs were also identified using the REBASE database of RM genes [10]. Alignments of identified homologs were performed using Clustal X2 [49]. Protein domain architecture and sequence features, including identification of Structural Classification of Proteins (SCOP) superfamilies, were analyzed using InterProScan [50].

Homologs to the CTAG modification methyltransferase in *H. volcanii* DS2 (ADE02643) in completely sequenced halobacterial genomes were identified using the NCBI's blast site for microbial genomes, selecting completely sequenced halobacterial genomes and the tblastn search algorithm. The list of completely sequenced genomes did not completely correspond to the genomes searched through the NCBI's web interface; therefore, the absence of a homolog in a genome was confirmed through a targeted tblastn search. The one additional homologous gene identified in this step was added to the phylogenetic analysis. Matching nucleotide sequences were retrieved, translated into protein and aligned using muscle [51] as implemented in Seaview [52], and used for phylogenetic reconstruction using PhyML [53] with the following parameters: LG substitution model, 100 bootstrap samples, 4 substitution rate categories with estimated Gamma distribution parameter, and estimated fraction of invariant sites, and a tree topology search using both Nearest Neighbor Interchange and Subtree Pruning and Regrafting. 

CTAG and GATC frequency and cumulative occurrence of these motifs were calculated with an in house Perl (Practical Extraction and Report Language) script.

### 2.6. Haloferax volcanii Growth Experiments

*Haloferax volcanii* strains H26 and *ΔRM* (Table 1) were grown in Hv-YPC medium to mid-log phase (OD_600_ = ~0.6–0.8). The cell cultures were then diluted in Hv-YPC to an OD_600_ of ~0.01 and were each distributed into 24 wells of a 96-well plate, with each well receiving 200 µL of culture. One well on the plate received 200 µL of Hv-YPC to be used as a blank reading. The 96 well plate was then covered with sealing tape and inserted into a Multiscan FC plate reader (Fisher Scientific, Waltham, MA, USA), which recorded the OD_620_ of each well every hour for 72 h while incubating and shaking the plate at 42 °C.

## 3. Results

### 3.1. Bioinformatics Analysis Supports Identification of HVO_0794 as a Chromosomal 4mC CTAG MTase

**Figure 1:**
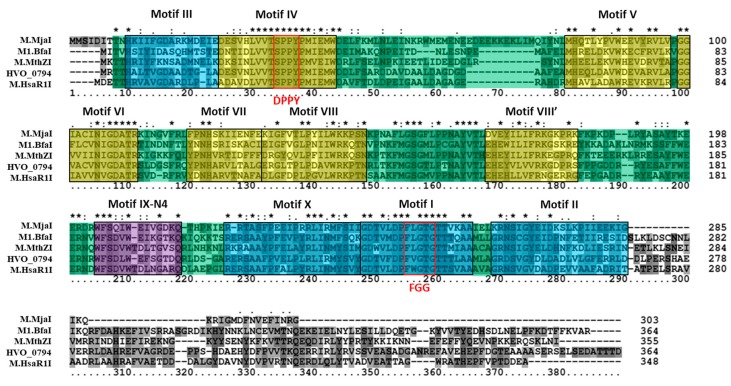
Amino acid alignment of HVO_0794 homologs. The multiple sequence alignment includes HVO_0794 (*Haloferax volcanii* DS2; GenBank ADE02643), M.HsaR1I (*Halobacterium salinarum* R1; GenBank CAP14114), M.MthZI (*Methanothermobacter thermautotrophicus*; GenBank CAA48447), M.MjaI (*Methanocaldococcus jannaschii* DSM 2661; GenBank AAB98988) and M.BfaI (*Bacteroides fragilis*; GenBank ADQ20483). Identified *N*4-cytosine methyltransferase motifs I-X [9] are highlighted in blue (representing *S*-adenosyl methionine (AdoMet) binding motifs), purple (representing DNA binding motif), and yellow (representing catalytic motifs). Red boxes are used to identify the signature DPPY and FGG motifs. The SCOP superfamily domain *S*-adenosyl-l-methionine-dependent methyltransferase domain SSF53335 is highlighted in green throughout the alignment. Clustal X2 shading and marking of amino acids is included in the alignment.

The putative 4mC CTAG MTase HVO_0794 was analyzed bioinformatically. A blastp analysis identified a homolog to the enzyme in *Methanothermobacter thermautotrophicus* named M.MthZI (GenBank CAA48447) which has been experimentally characterized as a 4mC CTAG MTase [33]. Two other homologs were also identified via blastp that were also experimentally characterized via unpublished work according to REBASE: M.BfaI (GenBank ADQ20483) in *Bacteroides fragilis* and M.MjaI (GenBank AAB98988) in *Methanocaldococcus jannaschii* DSM 2661. These homologs are similar in size to HVO_0794, ranging between 303 to 364 amino acids in length. Also, these enzymes are classified as Type II, subtype β 4mC MTases on REBASE, as is HVO_0794. A multiple sequence alignment (Figure 1) of HVO_0794 with these homologs and homolog M.HsaR1I (GenBank CAP14114) from *Halobacterium salinarium* R1 indicate significant sequence similarity is shared in the N-terminal and central regions of the amino acid sequences. This region of sequence similarity belongs to the *S*-adenosyl-l-methionine-dependent methyltransferase superfamily domain SSF53335 identified by InterProScan in the SCOP database. Signature *N*4-methyltransferase motifs PR00508 from the protein motif database PRINTS were also identified in the region via InterProScan (data not shown). A closer examination of the alignment revealed the presence of motifs I-X identified in M.MthZI and other 4mC MTases by Bujnicki and Radlinska [9]. These motifs are present in the alignment in the order of N-III-IV-V-VI-VII-VIII-VIII’-IX-X-I-II-C which is indicative of subtype β MTases [8]. The signature AdoMet binding motif DPPY and catalytic motif FGG are also fully conserved in the alignment (DPPY conserved here as SPPY). The FGG motif also occurs before the DPPY motif in the alignment, a motif order observed in subtype β MTases according to REBASE [10]. Overall, these results support the identification of HVO_0794 as a 4mC CTAG MTase of the subtype.

**Figure 2:**
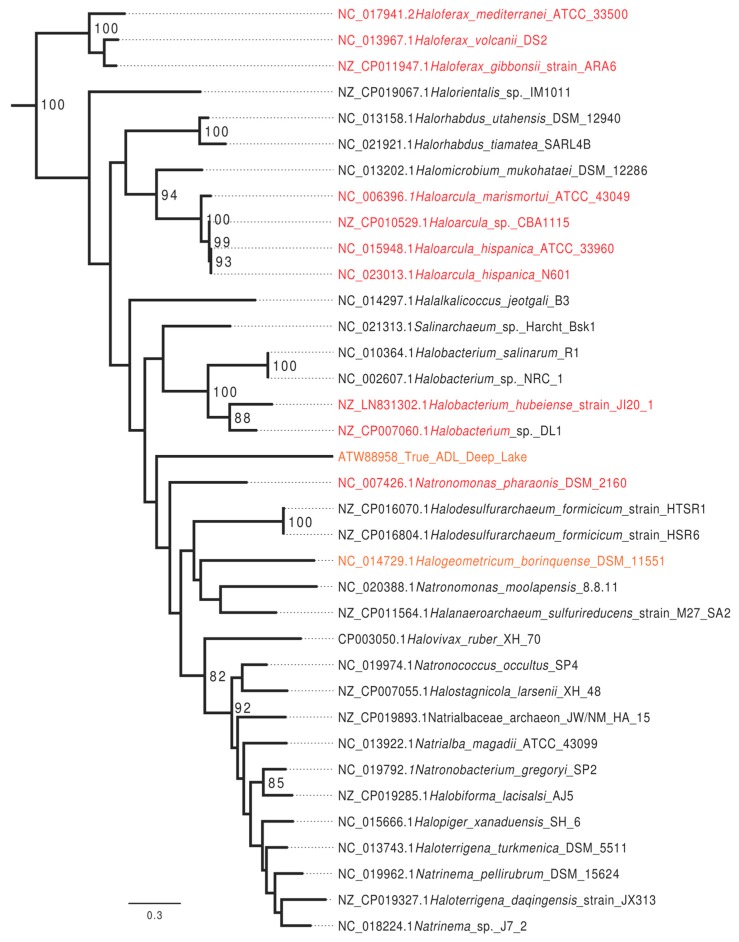
Maximum likelihood phylogeny of homologs of the *Haloferax volcanii* DS2 CTAG MTase identified in completely sequenced haloarchaeal genomes. Numbers give non-parametric bootstrap support values. The phylogeny was rooted using more divergent haloarchaeal and methanomicrobial homologs. Genomes with a chromosome wide GATC to CTAG ratio below five are given in red, those with a GATC to CTAG ratio between 5 and 14 are given in orange. In addition, those with a ratio above 20 are given in black. Note that only few groups are well supported, including the *Haloferax* and *Haloarcula* genera.

In a search of all completely sequenced halobacterial genomes available on 12 June 2017, homologs that group with HVO_0794 with high statistical support were identified in 37 (88%) of the completely sequenced genomes. Genomes with the homolog present are included in Figure 2. Homologs that grouped with the *H. volcanii* CTAG enzyme with high support were absent in *Halorubrum lacusprofundi* ATCC 49239, *Halorubrum trapanicum, Haloquadratum walsbyi* C23, *Haloquadratum walsbyi* DSM 16790, and *Halopenitus persicus*. 

**Figure 3:**
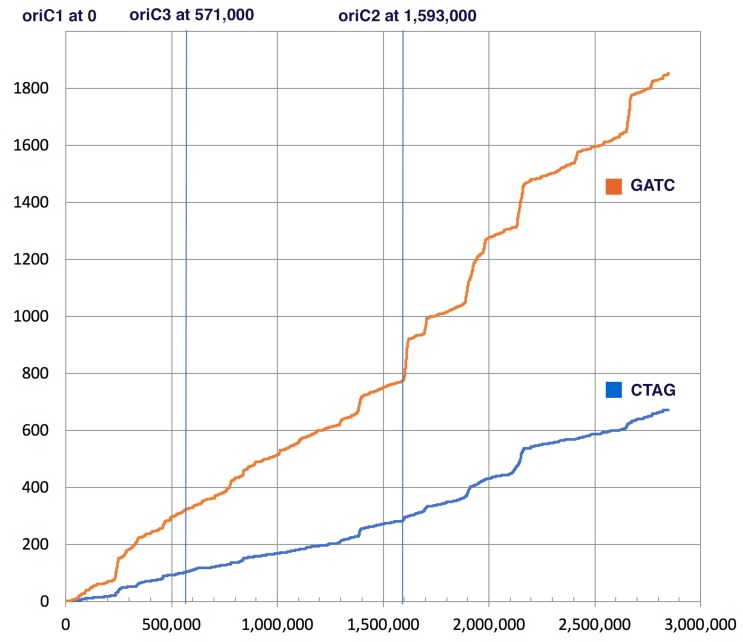
Cumulative occurrence (*y*-axis) of GATC (orange) and CTAG (blue) motifs along the *Haloferax volcanii* DS2 genome (*x*-axis). The location of the origins of replication identified in Hawkins et al. [54] are indicated on top.

The GATC motif is methylated in many organisms and this methylation was shown to play a role in regulating the start of replication and in mismatch repair of newly synthesized DNA strands [23,24,25,26,27]. CTAG and GATC motifs occur throughout the *H. volcanii* genome. In contrast to other Halobacteria, both motifs show localized areas of higher concentrations within the genome. The *H. volcanii* chromosome possesses several origins of replication [54], one of these (oriC2) is associated with an increased concentration of CTAG and GATC motifs (Figure 3). 

In *E. coli* and other organisms where GATC methylation facilitates recognition of the newly synthesized DNA strand during mismatch repair, the GATC motif occurs with higher frequency as compared to the CTAG motif (22 times in *E. coli*, 46 times in *H. trapanicum*, see Table 4). In *H. volcanii* this ratio is only 2.8 (see Figure 2 and Table 4). Ratios below 5 were found in other *Haloferax* species, *Haloarcula* sp., *Natronomonas pharaonic*, *Halobacterium hubeiense* strain JI20-1, and *Halobacterium* sp. DL1; whereas *Halobacterium salinarum* R1 has a ratio above 20 (Table 4). In *Haloferax* spp. this drop in relative GATC frequency is due to a dropin frequency of the GATC (Table 4). The CTAG motif actually occurs less frequently in *H. volcanii* (0.24 times per 1000 nucleotides) than in other halobacterial chromosomes (average ± standard deviation in all completely sequenced chromosomes is 0.42/kb (±0.19). 

### 3.2. Bioinformatics Analysis Supports Identification of RmeM as a Type I 6mA MTase and RmeS as a Type I Specificity Subunit on the Chromosome

**Figure 4:**
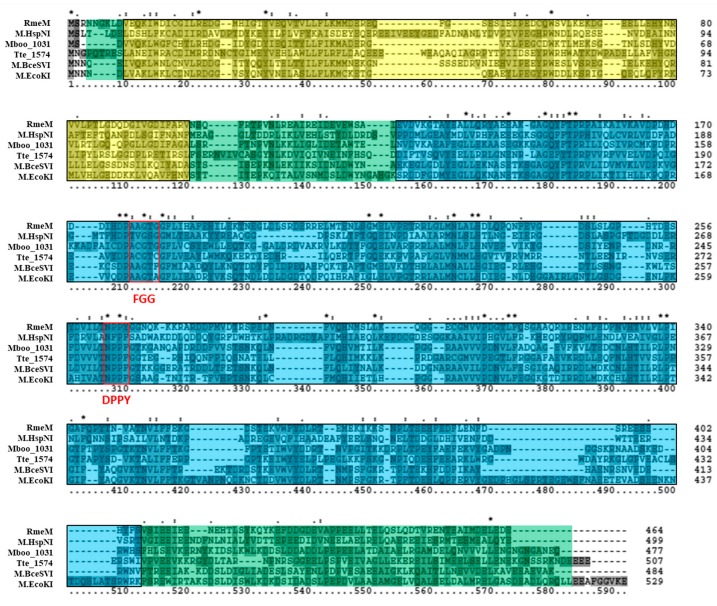
Amino acid alignment of RmeM homologs. The multiple sequence alignment includes RmeM (*Haloferax volcanii* DS2; GenBank ADE02452), M.HspNI (*Halobacterium salinarum* NRC-1; GenBank AAG18733), Mboo_1031 (*Methanoregula boonei* 6A8; GenBank ABS55549), Tte_1547 (*Caldanaerobacter subterraneus subsp. tengcongensis* MB4; AAM24756), M.BceSVI (*Bacillus cereus* ATCC 10987; GenBank AAS39772), and M.EcoKI (*Escherichia coli* K-12; GenBank P08957). The PFAM N6 adenine-specific DNA methyltransferase N-terminal domain PF12161 is highlighted in yellow, and the PFAM DNA methylase, adenine specific domain PF02384 is highlighted in blue. Red boxes are used to identify the signature DPPY and FGG motifs. The SCOP superfamily domain *S*-adenosyl-l-methionine-dependent methyltransferase domain SSF53335 is highlighted in green throughout the alignment. Clustal X2 shading and marking of amino acids is included in the alignment.

The putative Type I 6mA MTase RmeM and its cognate specificity subunit RmeS were also analyzed via bioinformatics. Tblastn of the RmeM sequence against the database of Halobacteria genomes in NCBI (taxid 183963) showed this MTase to be relatively rare in this Order, as we retrieved significant hits in 19 out of 181 (10.5%) genomes of Halobacteria, and 3 out of 42 (7.1%) of the fully sequenced genomes. Blastp analysis of RmeM indicated that it is homologous to M.EcoKI (GenBank P08957), a well-characterized Type I 6mA MTase in *E. coli* [55,56]. A homolog to RmeM was also identified in *Bacillus cereus* ATCC 10987 (M.BceSVI; GenBank AAS39772), which has been characterized via SMRT sequencing [57], as well as in *Methanoregula boonei* 6A8 (Mboo_1031; GenBank ABS55549) which was characterized via unpublished research according to REBASE. These enzymes are 477 to 529 amino acids in length, which is similar to RmeM in size. The classification on REBASE for these enzymes also matches RmeM (Type I, subtype γ MTase). A multiple sequence alignment (Figure 4) of these identified homologs, along with a homolog identified in *Caldanaerobacter subterraneus* subsp. *tengcongensis* MB4 (Tte_1547; GenBank AAM24756) and in *Halobacterium salinarum* NRC-1 (M.HspNI; GenBank AAG18733) indicated sequence similarity is shared throughout the alignment. The same SCOP superfamily domain SSF53335 present in the HVO_0794 homologs was also identified in these homologs and spans most of the alignment. InterProScan also identified PFAM protein family database domain PF12161, an N-terminal domain present in Type I MTases which affects the affinity of the MTase for hemimethylated DNA [58]. Another PFAM domain was also detected: PF02384, which is a 6mA MTase domain found in Type I MTase enzymes. The catalytic signature motif FGG is conserved (AGG in this alignment), although the third residue of the motif is not well-conserved in M.BceSVI and M.EcoKI. The AdoMet binding signature motif DPPY is also conserved in the alignment as NPP(Y/F). Both of these signature motifs are poorly conserved in M.HspNI. The order of these signature motifs in the alignment, with FGG occurring before DPPY, is typical of subtype γ MTases according to REBASE [10]. These data indicate that RmeM is a Type I, subtype γ MTase.

**Figure 5:**
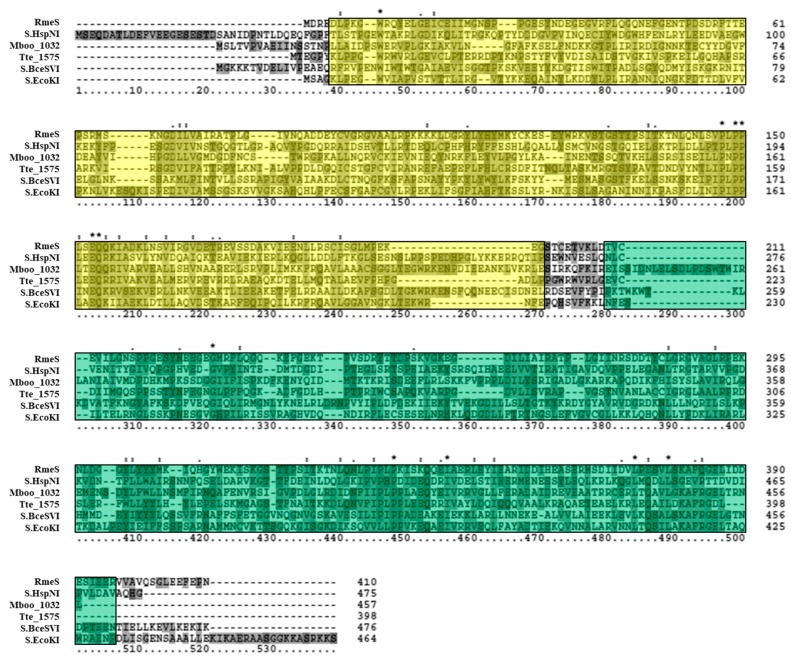
Amino acid alignment of RmeS homologs. The multiple sequence alignment includes RmeS (*Haloferax volcanii* DS2; GenBank ADE04051), S.HspNI (*Halobacterium salinarum* NRC-1; GenBank AAG18734), Mboo_1032 (*Methanoregula boonei* 6A8; ABS55550), Tte1545 (*Caldanaerobacter subterraneus* subsp. *tengcongensis* MB4; GenBank AAM24754), S.BceSVI (*Bacillus cereus* ATCC 10987; GenBank AAS39773), and S.EcoKI (*Escherichia coli* K-12; GenBank AAG18734). The first SCOP superfamily domain DNA methylase specificity domain SSF116734 is highlighted in yellow, and the second one is highlighted in green. Clustal X2 shading and marking of amino acids is included in the alignment.

Tblastn of the RmeS sequence against the NCBI database of Halobacteria genomes (taxid 183963) resulted in significant hits in 26 out of 181 (14.4%) genomes of Halobacteria, and 7 out of 42 (16.7%) of the fully sequenced genomes. Further analysis of RmeS via blastp and PSI-BLAST resulted in the identification of a homolog in *E. coli* K-12 called S.EcoKI (GenBank AAC77304), a well-characterized site specificity subunit that belongs to the same Type I RM system as M.EcoKI [59]. Blastp analysis also indicated that RmeS is homologous to Tte_1545 (GenBank AAM24754), a Type I site specificity subunit in *Caldanaerobacter subterraneus* subsp. *tengcongensis* MB4 which corresponds to the same RM system as Tte_1547 and has been structurally analyzed [60]. RmeS also shares homology with S.BceSVI (GenBank AAS39773), the site specificity subunit which belongs to the same Type I RM system as M.BceSVI in *Bacillus cereus* ATCC 10987 [57]. RmeS was also observed to be homologous to the putative cognate site specificity subunit of M.HspNI in *Halobacterium salinarum* NRC-1 (S.HspNI; GenBank AAG18734) as well as the putative site specificity subunit of Mboo_1031 in *Methanoregula boonei* 6A8 (Mboo_1032; GenBank ABS55550). These homologs range from 398 to 476 amino acids in length, similar to RmeS which is 410 amino acids long, and were all classified as Type I specificity subunits on REBASE. A multiple sequence alignment of these enzymes (Figure 5) did not show high sequence conservation among the homologs. However, InterProScan revealed that these homologs all shared the same SCOP superfamily DNA methylase specificity domain SSF116734. This superfamily domain was observed to occur twice in similar regions of the homologs in the alignment: one was more N-terminal in its location and the other was more C-terminal. Within these regions, the PFAM restriction endonuclease, type I, HsdS domains PF01420 were observed to occur (not shown in the alignment), which correspond to the two target recognition domains of Type I site-specificity subunits [61]. In summary, these results indicate that RmeS is the cognate Type I site-specificity subunit of RmeM.

### 3.3. Bioinformatics Analysis Supports Annotation of HVO_C0040 as a 5mC MTase and HVO_A0079 as a 6mA MTase

**Figure 6:**
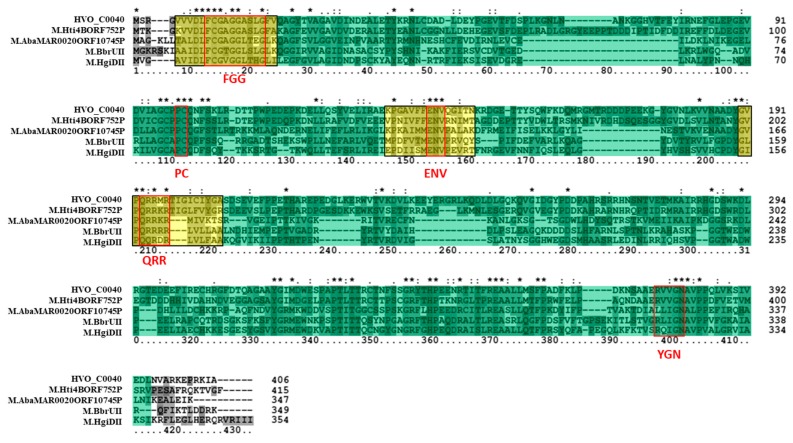
Amino acid alignment of HVO_C0040 homologs. The multiple sequence alignment includes HVO_C0040 (*Haloferax volcanii* DS2; GenBank ADE05226), M.Hti4BORF752P (*Halorhabdus tiamatea* SARL4B; GenBank CCQ33914), M.AbaMAR002ORF10745P (*Acinetobacter baumannii* MAR002; GenBank KGF60346), M.BbrUII (*Bifidobacterium breve* UCC2003; GenBank ABE95799), and M.HgiDII (*Herpetosiphon aurantiacus*; GenBank CAA38941). The protein motif database PRINTS cytosine-specific DNA methyltransferase signature domains PR00105 are highlighted in yellow. Red boxes are used to identify signature FGG, PC, ENV, QRR, and YGN motifs described in Posfai et al. [11]. The SCOP superfamily domain *S*-adenosyl-l-methionine-dependent methyltransferase domain SSF53335 is highlighted in green throughout the alignment. Clustal X2 shading and marking of amino acids is included in the alignment.

The other two putative MTase genes in *H. volcanii* DS2, which are located on plasmids, were also examined bioinformatically. Putative Type II 5mC MTase HVO_C0040 is located on extrachromosomal plasmid pHV1 and is flanked by an upstream IS4 family transposase (HVO_C0039). Tblastn of the HVO_C0040 sequence against the NCBI database of Halobacteria genomes (taxid 183963) resulted in significant hits in 87 out of 181 (48.1%) of halobacterial genomes, and 13 (31%) of the fully sequenced genomes. A blastp analysis of HVO_C0040 revealed that it is homologous to M.HgiDII (GenBank CAA38941) in *Herpetosiphon aurantiacus*, which has been experimentally characterized as a 5mC MTase recognizing GTCGAC [62]. HVO_C0040 was also observed to share homology with M.BbrUII (GenBank ABE95799), which has been characterized in *Bifidobacterium breve* UCC2003 as a 5mC MTase [63]. Two other homologs identified via blastp include the putative 5mC MTase in halobacterial species *Halorhabdus tiamatea* SARL4B (M.Hti4BORF752P; GenBank CCQ33914). Putative 5mC MTase in *Acinetobacter baumannii* MAR002 (M.AbaMAR002ORF10745P; GenBank KGF60346) was also identified as a homolog via blastp. These sequences range from 347 to 415 amino acids in length, which is similar to the 406-amino acid length of HVO_C0040. These sequences are also all annotated as Type II 5mC MTases on REBASE. An amino acid alignment of these homologs (Figure 6) indicates that sequence similarity is shared in many regions of the amino acid sequences. Many of these regions where significant sequence similarity is observed are identified as signature 5mC MTase motifs. Three regions of sequence similarity, for example, are identified by InterProScan as PRINTS cytosine-specific DNA MTase signature domains PR00105. Other regions match the 5mC conserved signature motifs identified by Posfai et al. [11], such as FGG, PC, ENV, QRR, and YGN (conserved here as (R/L)GN). Each sequence also belongs to the SCOP *S*-adenosyl-l-methionine-dependent MTase superfamily domain SSF53335 identified by InterProScan. Overall, these data support the annotation of HVO_C0040 as a 5mC MTase.

**Figure 7:**
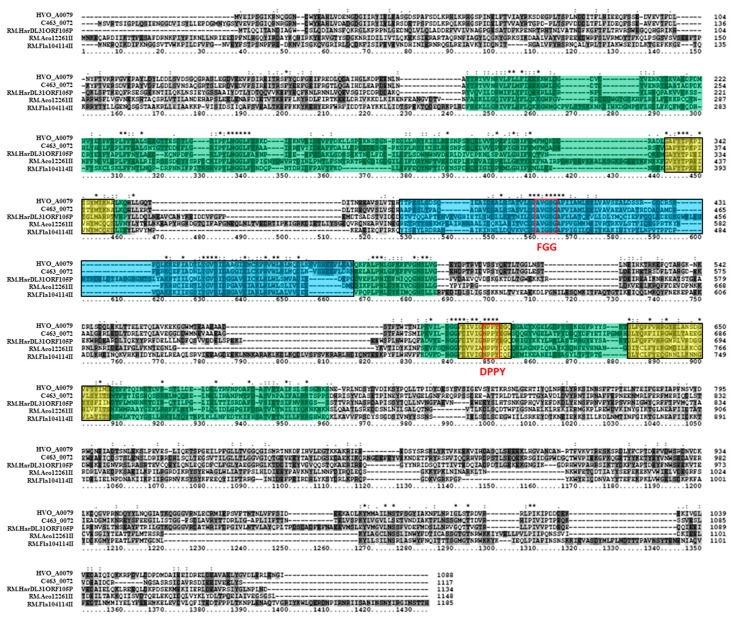
Amino acid alignment of HVO_A0079 homologs. The multiple sequence alignment includes HVO_A0079 (*Haloferax volcanii* DS2; GenBank ADE01706), C463_0072 (*Halorubrum californiensis* DSM 19288; GenBank ELZ48543), RM.HarDL31ORF105P (*Halophilic archaeon* DL31; GenBank AEN07377), RM.Aco12261II (*Aminobacterium colombiense* DSM 12261; GenBank ADE57453), and RM.Fla104114II (*Filimonas lacunae* 104114; GenBank BAV07385). The PRINTS adenine-specific DNA methyltransferase signature domains PR00507 are highlighted in yellow. Red boxes signify signature FGG and DPPY motifs. The SCOP superfamily domain *S*-adenosyl-l-methionine-dependent methyltransferase domain SSF53335 is highlighted throughout the alignment. Clustal X2 shading and marking of amino acids is included in the alignment.

Putative Type IIG 6mA MTase HVO_A0079 is located on extrachromosomal plasmid pHV4 and is flanked by a downstream IS4 family transposase (HVO_A0080). Tblastn of the HVO_A0079 sequence against the NCBI database of halobacterial genomes (taxid 183963) resulted in significant hits in 101 out of 181 (59.1%) genomes of Halobacteria, and 21 (50%) of the fully sequenced genomes, indicating a higher prevalence in the Order than the RmeM or RmeS homologs, but not as high as HVO_0794 homologs. Blastp analysis of putative Type IIG 6mA MTase HVO_A0079 identified a number of homologs to the protein, including RM.Aco12261II (GenBank ADE57453), a Type IIG 6mA MTase in *Aminobacterium colombiense* DSM 12261 which has been identified via SMRT sequencing as targeting the motif CCRGA^m6^G [32]. HVO_A0079 was also observed to share homology with RM.Fla104114II (GenBank BAV07385), a Type IIG 6mA MTase characterized via unpublished SMRT sequencing data on REBASE. Blastp also determined that HVO_A0079 shared homology with putative Type IIG 6mA MTases in *Halorubrum californiensis* DSM19288 (C463_0072; GenBank ELZ48543) and in *Halophilic archaeon* DL31 (RM.HarDL31ORF105P; GenBank AEN07377). These proteins range between 1117 to 1185 amino acids in length, which is similar to the 1088 amino acid length of HVO_A0079. These homologs are also all annotated on REBASE as Type IIG 6mA subtype α RM proteins, with the exception of C463_0072, which is not present in REBASE. A multiple sequence alignment of the amino acid sequences of these homologs (Figure 7) indicated significant sequence conservation in the central region of the alignment. Three large sections of this central region were observed via InterProScan to belong to the SCOP *S*-adenosyl-l-methionine-dependent methyltransferase superfamily domain SSF53335. InterProScan also identified three regions of the alignment which belong to the PRINTS adenine-specific DNA MTase signature domains PR00507, as well as a PFAM Eco57I domain PF07669, a domain observed in well-characterized Type IIG RM protein Eco57I [64]. A closer analysis of the alignment revealed the presence of FGG (conserved as AGG) and DPPY (conserved as NPPY) signature motifs in the order N-FGG-NPPY-C, which would follow the motif order N-FGG-TRD-DPPY-C observed in subtype α MTases according to REBASE [10]. However, no significant similarity was observed in the N-terminal region of these proteins, which is where the restriction endonuclease domain is typically located in Type IIG RM proteins. These results overall support the annotation of HVO_A0079 as a Type II 6mA subtype α MTase.

### 3.4. Deletion of HVO_0794, HVO_A0006, and HVO_A0237 Eliminates 4mC Methylation and Does Not Effect 6mA Methylation

In order to better understand the roles of HVO_0794, HVO_A0006, and HVO_A0237 in DNA methylation, the three genes were deleted in *mrr* deletion strain H1206, producing a triple deletion mutant (∆*HVO_0794* ∆*HVO_A0006* ∆*HVO_A0237*). The genome of this deletion mutant was sequenced via SMRT sequencing to determine the methylome and the results are listed in Table 5. In this strain, the C^m4^TAG motif that is modified in the parental strain H26 [37] is no longer detected as methylated. Also, the 6mA motif GCA^m6^BN_6_VTGC is modified in the triple deletion mutant, with 100% of the 410 motifs in the genome identified as methylated. Between studies, there was also a difference in the percent of motifs detected as methylated in H26 compared to the triple deletion mutant. In H26, only 316 of the 410 GCABN_6_VTGC motifs (~77%) were detected as methylated in [37], whereas in this study all 410 motifs are modified in ∆*HVO_0794* ∆*HVO_A0006* ∆*HVO_A0237*. This discrepancy is likely the result of a difference in sequence coverage, since the mean motif coverage and QV scores (confidence scores) were greater in the triple deletion mutant compared to H26. In ∆*HVO_0794* ∆*HVO_A0006* ∆*HVO_A0237*, the mean motif coverage for GCA^m6^BGN_5_VTGC was 130.4, a ~325% increase from the mean motif coverage of 30.7 in H26. The mean modification QV score for the 6mA motif was 213.0 in the triple deletion mutant, an increase of ~274% from the H26 mean QV score of 57.0. Therefore, it is likely that the motifs were methylated completely in both strains, but that some of those motifs were not detected as modified in H26 due to the lower coverage and mean QV scores. Overall, these results indicate that deletion of *HVO_0794*, *HVO_A0006*, and *HVO_A0237* abolishes methylation of C^m4^TAG, but has no effect on methylation of GCA^m6^BN_6_VTGC.

### 3.5. Deletion of the rmeRMS Operon Abolishes 6mA Methylation

The putative Type I operon *rmeRMS* was deleted in *H. volcanii* H1206, and sequenced via SMRT sequencing, in order to determine the role of the operon in DNA methylation. The results of the SMRT analysis for this strain (∆*rmeRMS*) are listed in Table 5. In ∆*rmeRMS*, the 6mA motif GCA^m6^BN_6_VTGC is not detected as modified as it is in H26, and no other 6mA methylation is present [37]. Modification of C^m4^TAG is still detected in the deletion strain. In ∆*rmeRMS*, 1199 of the 1342 CTAG motifs in the genome (~89%) are detected as methylated, These results are better than in Ouellette et al. [37] due to the better sequence coverage in ∆*rmeRMS* compared to H26, thus providing better detection of the methylated motifs. The mean motif coverage for C^m4^TAG in this deletion mutant was 113.0. The mean modification QV score for the 4mC motif in ∆*rmeRMS* was 104.1. Overall, these results indicate that deletion of the *rmeRMS* operon eliminates methylation of the GCA^m6^BN_6_VTGC motif.

### 3.6. Multi-RM Deletion Eliminates Detection of All DNA Methylation

A multi-RM deletion mutant, with all putative RM genes except for *HVO_C0040* deleted from the strain, was also analyzed using SMRT sequencing to determine if the deletion of these genes resulted in elimination of DNA methylation in *H. volcanii*. The results of the SMRT analysis for this strain (∆*RM*) are listed in Table 5. The 6mA motif GCA^m6^BN_6_VTGC identified in H26 is not detected as modified in this strain [37]. Also, the 4mC motif C^m4^TAG is also not detected as methylated. No other motifs are detected as modified in this strain. These results indicate that all DNA methylation that can be detected by SMRT sequencing has been eliminated in the multi-RM deletion mutant. Although the remaining RM gene in this strain (*HVO_C0040*) encodes a MTase predicted to perform 5mC methylation which is difficult to detect via SMRT sequencing without Tet treatment [65], motifs of this type of methylation can still be weakly detected without Tet treatment. Since even weak detection of motifs was not observed in this strain, the results indicate that HVO_C0040 is not active as an MTase.

### 3.7. No Defect in Growth Occurs in the Multi-RM Deletion Compared to the Parental Strain

**Figure 8:**
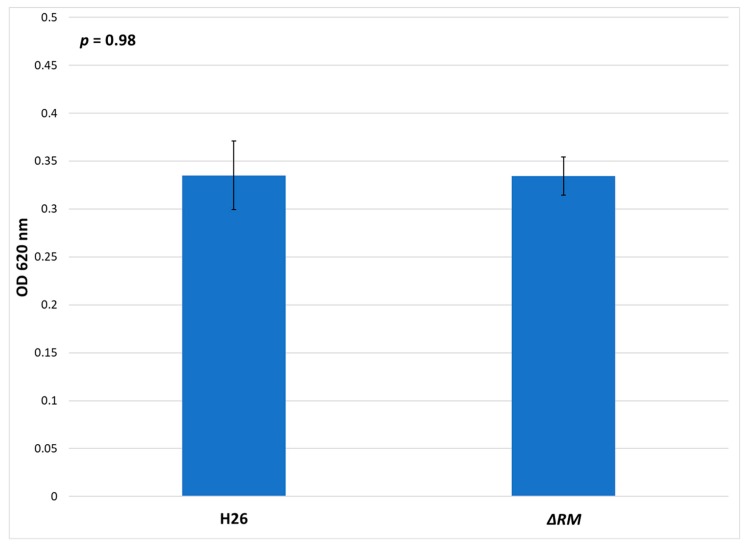
Cell density of H26 and *ΔRM* at stationary phase when grown on Hv-YPC, represented by the average optical density (OD_620_) reading of 24 cell culture replicates after 72 h of growth. Error bars indicate the standard error of the mean. Analysis of variance (ANOVA) single factor, *p* = 0.98.

In *E. coli dam^−^* mutants, the lack of methylation results in growth defects compared to the wild-type strain [66]. In order to determine if the lack of RM genes resulted in a deficiency of growth in the *ΔRM* strain compared to the H26 parental strain, both strains were grown in Hv-YPC medium (Figure 8). The results indicate that no significant difference in growth. Both strains entered log phase at ~6 h, and although *ΔRM* initially had a slightly higher OD_620_ when it entered log phase, this difference disappeared after ~20 h of growth, and both cultures reached stationary phase at ~36 h with similar OD_620_ values (Supplementary Figure S1). The final OD_620_ at for H26 after 72 h was 0.335, whereas for *ΔRM* the final OD_620_ was 0.334. The difference between these two averages was not significant based on the standard error values and analysis of variance (ANOVA) single factor statistical analysis. Overall, these results indicate that there is no detectable defect in growth in the *ΔRM* strain compared to the H26 strain.

## 4. Discussion

In a previous study on DNA methylation in *H. volcanii* H26 [37], two motifs were identified as modified throughout the genome of the organism: the 4mC motif C^m4^TAG and the 6mA motif GCA^m6^BN_6_VTGC. These motifs were predicted to be methylated by a putative Type II 4mC MTase encoded by *HVO_0794* and a putative 6mA MTase belonging to a Type I RM system encoded by the operon *HVO_2269-2271* (*rmeRMS*), respectively. However, there are several annotated RM genes with no predicted motif recognition. In this follow-up study, we demonstrated through successive deletions of annotated RM genes that the C^m4^TAG motif is methylated by the Type II MTase HVO_0794; the GCA^m6^BN_6_VTGC motif is methylated by the Type I RM system RmeRMS; and that the other annotated MTases do not methylate under the conditions tested.

In mutants with *HVO_0794* deleted from the genome, the SMRT sequencing analyses did not detect methylation of C^m4^TAG or any other type of 4mC methylation, indicating that the MTase encoded by this gene is responsible for CTAG methylation since removal of this gene abolishes methylation of the motif. This result confirms predictions from previous studies [47,67] that suggested HVO_0794 is a CTAG MTase. Our bioinformatics analysis also supports the identification of this MTase as responsible for 4mC methylation, since the amino acid sequence has high similarity to previously characterized Type II 4mC CTAG MTases such as M.MthZI [33]. Although *HVO_A0006* and *HVO_A0237* were also deleted in the same strain as *HVO_0794*, neither of these genes have high similarity to 4mC CTAG MTases, and deletion of *HVO_A0006* in a previous study [37] indicated that it does not affect cytosine methylation, ruling out these genes as candidates for C^m4^TAG methylation. Based on our search of REBASE and NCBI, no cognate REase is encoded in the genome of *H. volcanii*, and deletion of the gene was not lethal as would be expected if there was a cognate REase, suggesting that HVO_0794 is an orphan MTase. The observation of an orphan CTAG MTase in *H. volcanii* was not unexpected based on previous work by Blow et al. [32], who found that predicted Type II CTAG orphan MTase gene families are common in the Halobacteria, occurring in 78% of halobacterial species. 

Several of the halobacterial species examined by Blow et al. [32] which contained the CTAG MTase family also had a high CTAG motif density at their origins of replication, suggesting that this gene family may play a role in regulating DNA replication in the Halobacteria. Our analysis of *H. volcanii* showed a higher CTAG motif density surrounding oriC2, but not in regions near the other two origins; however, the oriC2 region also showed the enrichment of GATC motifs was even more pronounced (see Figure 3). It remains to be established, if the stretches with higher CTAG and GATC motif density have a selected function in *H. volcanii*, or if they reflect gene acquisition from a donor with different compositional bias. 

*H. volcanii* has a lower GATC to CTAG ratio than organisms with homologs to the *E.coli* DNA adenine methyltransferase (Dam) which recognizes the GATC motif, and aids DNA repair via a methyl-directed mismatch repair system [21]. However, the decrease in the GATC to CTAG ratio in *H. volcanii* is due exclusively due to a drop in the frequency of the GATC motif, and not to an increase in the CTAG frequency. The role of CTAG methylation may not be of major importance for *H. volcanii*, as no growth defect was observed to occur in the *ΔRM* strain compared to the parental H26 strain. Considering that *H. volcanii* does not require origins of replication in order to grow efficiently [54], it is not too surprising that eliminating the putative role of HVO_0794 in regulating the origins of replication does not affect growth.

The absence of GCA^m6^BN_6_VTGC methylation in deletion mutants without the *rmeRMS* operon indicated that these genes are responsible for 6mA methylation in *H. volcanii*. Our bioinformatics analysis also indicates that RmeRMS is a Type I RM system, since both the MTase subunit RmeM and specificity subunit RmeS are homologous to well-characterized Type I 6mA MTases and specificity subunits such as M.EcoKI and S.EcoKI [56]. The motif GCA^m6^BN_6_VTGC resembles the type of sequences targeted by Type I systems, which are typically bipartite sequences with a gap of unspecified nucleotides in the middle [15]. Therefore, the observation that *rmeRMS* is a Type I RM system supports the identification of this operon as responsible for 6mA methylation in *H. volcanii*. Previous work by Ouellette et al. [37] had suggested that the RM gene *HVO_A0006* might have a role in 6mA methylation, since SMRT sequencing of a *HVO_A0006* deletion mutant identified an alteration in the 6mA motif (GCA^m6^BGN_5_VTGC instead of GCA^m6^BN_6_VTGC). However, our SMRT sequencing analysis of a deletion mutant without *HVO_A0006* (∆*HVO_0794* ∆*HVO_A0006* ∆*HVO_A0237*) did not demonstrate any difference in the 6mA motif compared to the H26 parental strain. This difference is likely due to better sequence coverage in our data (~400x coverage for ∆*HVO_0794* ∆*HVO_A0006* ∆*HVO_A0237* compared to ~80x coverage for *ΔHVO_A0006*), allowing our analysis to identify more motifs as modified in the genome compared to the previous study [37]. This result, along with the observation that deletion of *rmeRMS* alone abolished detection of 6mA methylation, suggests that RmeRMS is solely responsible for adenine methylation in *H. volcanii*. 

The presence of a restriction-subunit encoding gene (*rmeR*) indicates that the system can also cleave DNA at unmethylated target motifs, acting as a fully functional RM system. It is possible that this system functions in protecting *H. volcanii* from foreign DNA similar to RM systems in other organisms [68]. This defense system, in combination with clustered regularly interspaced short palindromic repeats (CRISPR-Cas system) [69,70], is likely advantageous to *H. volcanii* considering that haloarchaeoviruses are highly abundant in hypersaline environments [71,72]. RmeRMS may also be involved in regulating gene transfer, which in *H. volcanii* can occur within species as well as between species [73]. In *E. coli*, for example, the Type I RM system EcoKI has been demonstrated to reduce uptake via conjugation of unmethylated plasmids with EcoKI target sites [74]. A study by Lin et al. [75] indicated that RM systems could limit the size of DNA fragments that can recombine in *Helicobacter pylori*. Correlation between RM system occurrence and phylogenetic clusters was observed in *Haemophilus influenzae*, suggesting that RM systems are acting as barriers to genetic exchange between phylogenetic groups [76]. RM systems have also been hypothesized to drive population dynamics and diversification in *Neisseria meningitidis* [77]. Our results also indicate that homologs to RmeRMS, as well as the other predicted RM genes, do not occur as frequently in haloarchaeal species compared to the CTAG orphan MTase family genes; the RmeRMS system could possibly limit gene transfer that occurs with other individuals in the environment which lack the system, thus acting as a barrier to recombination for *H. volcanii*.

Our SMRT sequencing analyses indicate that deletion of *HVO_0794* and *rmeRMS* is sufficient to eliminate detection of methylation in *H. volcanii*, indicating that the other predicted MTase genes in the organism (*HVO_C0040*, *HVO_A0079*, and *HVO_A0237*) do not contribute to methylation. The reason for the apparent inactivity of these genes is unclear, considering that our bioinformatics analyses indicate that these genes share homology with characterized MTase genes in other organisms. Inactive RM genes have been observed to occur in other organisms, such as those belonging to the MmeI RM gene family [78]. These inactive genes can be readily reactivated, and were hypothesized to exist in a population to confer a selective advantage to individuals when the population undergoes disruption from foreign parasitic DNA [78]. However, these MmeI RM genes were inactivated as a result of disruptive mutations which do not appear to be present in the predicted RM genes in *H. volcanii*. It is possible that these genes may still be active in *H. volcanii* but are only expressed under conditions not tested. However, a blastn search of the *H. volcanii* DS2 transcriptome data from Babski et al. [79] (sequence read archive (SRA) accession number SRP076059) using these three genes as queries suggested that they are expressed, although the search results do not indicate if functional protein products of these genes are produced. Interestingly, our results indicate that HVO_C0040 and HVO_A0079 are flanked by transposase genes similarly to HVO_A0237 [37]. Perhaps these genes are mobile genetic elements, as is the case with many RM genes [80], and they became non-functional when transferred into *H. volcanii*. Nevertheless, HVO_C0040, HVO_A0079, and HVO_A0237 do not appear to contribute to the methylome of *H. volcanii* under standard growing conditions. A possible exception to this list is HVO_C0040, the only remaining putative MTase gene in our *ΔRM* strain, which our bioinformatics analysis indicates is a 5mC MTase. Methylation patterns produced from 5mC MTases are typically difficult to detect with SMRT sequencing in the absence of Tet treatment [40,65]. However, 5mC methylation usually produces some signal via SMRT sequencing, yet we did not detect it in any of our multiple analyses including of the null mutant, leading us to think it is not methylating.

We also report in this study the construction of a MTase null mutant in *H. volcanii*. This strain (*ΔRM*) has all putative RM genes deleted from the genome with the exception of *HVO_C0040*, and our SMRT sequencing analysis indicates that this strain has no genomic methylation. We anticipate that this strain will be useful for future studies that examine the impact of RM systems and DNA methylation on cellular processes in *H. volcanii*, in which the *ΔRM* strain can be compared to the parental strain H26 that has all the RM genes intact. This strain could also be useful for characterizing putative MTase genes in other halobacterial strains via gene knock-in and SMRT sequencing to determine the target sites for methylation. We expect that this strain will be a useful tool in the quest to better understand DNA methylation and RM systems in the Halobacteria and other archaeal organisms.

## Figures and Tables

**Table 1:** Strains and plasmids used in this study.

**Strain/Plasmid Name**	**Description**	**Source**
*E. coli* HST08	Cloning strain of *E. coli*	Clontech, Cat. # 636763
*H. volcanii* DS2	Wild-type strain	Mullakhanbhai and Larsen [43]
*H. volcanii* H26	∆*pyrE2*; uracil auxotroph derived from DS2	Bitan-Banin et al. [44]
*H. volcanii* H1206	∆*pyrE2*/∆*mrr*; derived from H26	Allers et al. [45]
*H. volcanii* ∆*rmeRMS*	*rmeRMS* deletion strain; derived from H1206	This study
*H. volcanii* ∆*HVO_0794* ∆*HVO_A0006* ∆*HVO_A0237*	Deletion strain of *HVO_0794*, *HVO_A0006*, and *HVO_A0237*; Derived from H1206	This study
*H. volcanii* ∆RM	Deletion strain of *HVO_0794*, *rmeRMS*, *HVO_A0006*, *HVO_A0074*, *HVO_A0079*, and *HVO_A0237*; derived from H1206	This study
pTA131	Vector used for gene deletion. Contains *lacZ* cloning site for blue-white screening, *ampR* ampicillin resistance gene for selectivity in *E. coli*, and *pyrE2* for screening in *H. volcanii*.	Allers, Ngo, Mevarech and Lloyd [41]
p∆*HVO_A0006*	Derivative of pTA131 with flanking regions of *HVO_A0006* inserted into *lacZ* cloning site for gene deletion	Ouellette, Jackson, Chimileski and Papke [37]
p∆*HVO_0794*	Derivative of pTA131 with flanking regions of *HVO_0794* inserted into *lacZ* cloning site for gene deletion	This study
p∆*rmeRMS*	Derivative of pTA131 with flanking regions of *rmeRMS* operon inserted into *lacZ* cloning site for gene deletion	This study
p∆*HVO_A0074*	Derivative of pTA131 with flanking regions of *HVO_A0074* inserted into *lacZ* cloning site for gene deletion	This study
p∆*HVO_A0079*	Derivative of pTA131 with flanking regions of *HVO_A0079* inserted into *lacZ* cloning site for gene deletion	This study
p∆*HVO_A0237*	Derivative of pTA131 with flanking regions of *HVO_A0237* inserted into *lacZ* cloning site for gene deletion	This study

**Table 2:** List of restriction-modification (RM) genes annotated in *Haloferax volcanii* DS2.

Gene Locus Tag	Gene Symbol	Putative RM Classification	Gene Size (bp)	Location in the Genome	Notes
*HVO_0682*	*mrr*	Type IV	1005	Chromosome	Type IV restriction endonuclease
*HVO_0794*	*zim*	Type II	1095	Chromosome	Putative 4mC CTAG methyltransferase
*HVO_2269-2271*	*rmeRMS*	Type I	2223, 1395, 1233	Chromosome	Operon which contains a putative Type I RM system with 6mA methyltransferase
*HVO_C0040*	-	Type II	1221	pHV1	Putative 5mC GTCGAC methyltransferase
*HVO_A0006*	-	Type IIG	660	pHV4	Putative restriction endonuclease fragment of HVO_A0237 [37]
*HVO_A0074*	-	Type IV	3315	pHV4	Putative Type IV restriction endonuclease
*HVO_A0079*	-	Type IIG	3267	pHV4	Putative 6mA Type IIG RM protein
*HVO_A0237*	-	Type IIG	2199	pHV4	Putative 6mA methyltransferase and target recognition protein

**Table 3:** List of primers used in this study.

Primer Name	Primer Sequence	Primer Description
*HVO_A0006 FR1F*	5’- CGG GCC CCC CCT CGA GTC AAG CAG TAC CTC AAC ACG GAA CA -3’	Used to amplify the flanking regions of *HVO_A0006* for insertion into pTA131 linearized with *Xho*I and *Xba*I (Primer designs from Ouellette et al. [37])
*HVO_A0006 FR1R*	5’- ATT CGA TAT CAA GCT GTC CTC AAG GAC GGC CTG CA -3’	
*HVO_A0006 FR2F*	5’- GAC GCG TTG ATA TCC CGA AGA ATC CAG TTG CTG TCT GTT G -3’	
*HVO_A0006 FR2R*	5’- GGA TAT CAA CGC GTC GGC ATT ATG CAA TTC -3’	
*HVO_0794 FR1F*	5’- GCT TGA TAT CGA ATT CCC CGC GAG AAA GAC GAG AAG -3’	Used to amplify the flanking regions of *HVO_0794* for insertion into pTA131 linearized with *Eco*RI and *Bam*HI
*HVO_0794 FR1R*	5’- GCC TGG TAG AAT TCC CGT ACG GAC GTA TTT CCC CCG A -3’	
*HVO_0794 FR2F*	5’- GGA ATT CTA CCA GGC CGA CGA CGA CCG ACT GAG GTC -3’	
*HVO_0794 FR2R*	5’- TAG AAC TAG TGG ATC CGA ACG GCA GCA CCC GCG A -3’	
*rmeRMS FR1F*	5’- CGG GCC CCC CCT CGA GTC GGT GTT TCG CAG GTC ATT C -3’	Used to amplify the flanking regions of the *rmeRMS* operon for insertion into pTA131 linearized with *Xho*I and *Cla*I
*rmeRMS FR1R*	5’- GGG CGC CAT CCA GGC TAC TCA CTA TAT TTC ACT CGG GGT A -3’	
*rmeRMS FR2F*	5’- GCC TGG ATG GCG CCC CTC ACC TAT TCA CAA AGA GAG GAA -3’	
*rmeRMS FR2R*	5’- ATA TCA AGC TTA TCG ATT GCC GGG TTT CCT GTT ATT TT CT -3’	
*HVO_A0074 FR1F*	5’ GCT TGA TAT CGA ATT CTG CTC GTC GTG GTA CTT GTC -3’	Used to amplify the flanking regions of *HVO_A0074* for insertion into pTA131 linearized with *Eco*RI and *Xba*I
*HVO_A0074 FR1R*	5’- CGG TAC CGA CAT GTT ATC TCA ATG CAG CGC TTC TC -3’	
*HVO_A0074 FR2F*	5’- AAC ATG TCG GTA CCG TTG AGG ACT GGG AGC GTA TC -3’	
*HVO_A0074 FR2R*	5’- TGG CGG CCG CTC TAG TTG AAG GTC TGT GTC GCA TC -3’	
*HVO_A0079 FR1F*	5’- GCG AAT TGG GTA CCG GCC CCG ACC TGC CTT GG -3’	Used to amplify the flanking regions of *HVO_A0079* for insertion into pTA131 linearized with *Apa*I and *Eco*RV
*HVO_A0079 FR1R*	5’- GCC TGG TAG AAT TCC CCG TGT TCG GTT AAG CGG A -3’	
*HVO_A0079 FR2F*	5’- GGA ATT CTA CCA GGC AAT GGG ATC TGA CGA AGG AGG -3’	
*HVO_A0079 FR2R*	5’- CTG CAG GAA TTC GAT CAT AAA GGT CTT CTC AGC GGT T -3’	
*HVO_A0237 FR1F*	5’- CGG GCC CCC CCT CGA GGT TCG CGC TCT TGC TCA GGT -3’	Used to amplify the flanking regions of *HVO_A0237* for insertion into pTA131 linearized with *Xho*I and *Xba*I
*HVO_A0237 FR1R*	5’- GGG ATC CAA AGC TTG AGG CGT TGC TGA CAT TAT ATC GAA G -3’	
*HVO_A0237 FR2F*	5’- CAA GCT TTG GAT CCC GCC TTT CTG CTG GCG AGT TTC C -3’	
*HVO_A0237 FR2R*	5’- TGG CGG CCG CTC TAG AAT ATC GCG CAG CTC TAT CGG G -3’	
*M13(-21) F*	5’- GTA AAA CGA CGG CCA GT -3’	Used for amplifying the multiple cloning site of pTA131 for screening
*M13 R*	5’- AGG AAA CAG CTA TGA CCA T -3’	

**Table 4:** CTAG and GATC motif frequencies in completely sequenced halobacterial chromosomes. Data for *Escherichia coli* K12 are given for comparison.

Accession Number, Organism and Chromosome Number	Total CTAG	Total GATC	CTAG/kb	GATC/kb	GATC/CTAG	Match to *E. coli* Dam $
NC 013967.1 *Haloferax volcanii* DS2	671	1851	0.24	0.65	2.8	
NZ CP007551.1 *Haloferax mediterranei* ATCC 33500	1130	1472	0.38	0.50	1.3	
NZ CP011947.1 *Haloferax gibbonsii* strain ARA6	556	1510	0.19	0.51	2.7	
NC 017941.2 *Haloferax mediterranei* ATCC 33500	1142	1500	0.39	0.51	1.3	
NC 023013.1 *Haloarcula hispanica* N601 chr.1	1497	7523	0.50	2.50	5.0	
NC 023010.2 *Haloarcula hispanica* N601 chr.2	340	1675	0.94	4.61	4.9	
NZ CP010529.1 *Haloarcula* sp. CBA1115	1849	9333	0.54	2.73	5.0	
NC 006396.1 *Haloarcula marismortui* ATCC 43049 chr.I	1816	6564	0.58	2.10	3.6	
NC 006397.1 *Haloarcula* marismortui ATCC 43049 chr.II	274	1011	0.95	3.51	3.7	
NC 015948.1 *Haloarcula hispanica* ATCC 33960 chr.I	1493	7462	0.50	2.49	5.0	
NC 015943.1 *Haloarcula hispanica* ATCC 33960 chr.II	479	2210	0.98	4.52	4.6	
NZ LN831302.1 *Halobacterium hubeiense* strain JI20-1	795	1820	0.32	0.72	2.3	
NZ CP007060.1 *Halobacterium* sp. DL1	1168	4634	0.41	1.63	4.0	
NC 002607.1 *Halobacterium* sp. NRC-1	551	11047	0.27	5.48	20.0	
NC 010364.1 *Halobacterium salinarum* R1	537	10991	0.27	5.49	20.5	
NC 012029.1 *Halorubrum lacusprofundi* ATCC 49239 chr.1	756	25016	0.28	9.15	33.1	+
NC 012028.1 *Halorubrum lacusprofundi* ATCC 49239 chr.2	389	3306	0.74	6.29	8.5	+
NC 007426.1 *Natronomonas pharaonis* DSM 2160	1016	1839	0.39	0.71	1.8	
NC 008212.1 *Haloquadratum walsbyi* DSM 16790	2290	14449	0.73	4.61	6.3	
NC 017459.1 *Haloquadratum walsbyi* C23	2281	14681	0.72	4.66	6.4	
NC 014729.1 *Halogeometricum borinquense* DSM 11551	1085	8407	0.38	2.98	7.7	
CP024845.1 Halophilic archaeon True-ADL	1786	23542	0.54	7.07	13.2	
NC 021921.1 *Halorhabdus tiamatea* SARL4B	892	27010	0.32	9.59	30.3	+
NC 013158.1 *Halorhabdus utahensis* DSM 12940	964	32101	0.31	10.30	33.3	
NC 013202.1 *Halomicrobium mukohataei* DSM 12286	918	27978	0.30	8.99	30.5	
NC 013743.1 *Haloterrigena turkmenica* DSM 5511	1347	37472	0.35	9.64	27.8	
NC 013922.1 *Natrialba magadii* ATCC 43099	1592	25139	0.42	6.70	15.8	
NC 014297.1 *Halalkalicoccus jeotgali* B3	1106	29489	0.39	10.50	26.7	
NC 015666.1 *Halopiger xanaduensis* SH-6	1090	33560	0.30	9.15	30.8	
NC 018224.1 *Natrinema* sp. J7-2	1393	33801	0.38	9.14	24.3	
NC 019792.1 *Natronobacterium gregoryi* SP2	2330	31628	0.62	8.35	13.6	
NC 019962.1 *Natrinema pellirubrum* DSM 15624	1384	36667	0.37	9.67	26.5	
NC 019964.1 *Halovivax ruber* XH-70	1256	30664	0.39	9.51	24.4	
NC 019974.1 *Natronococcus occultus* SP4	1534	42563	0.38	10.61	27.7	
NC 020388.1 *Natronomonas moolapensis* 8.8.11	1088	23003	0.37	7.90	21.1	
NC 021313.1 *Salinarchaeum* sp. Harcht-Bsk1	948	33056	0.29	10.15	34.9	
NZ AP017558.1 *Halopenitus persicus* DNA CBA1233	695	31995	0.23	10.78	46.0	+
NZ AP017569.1 *Halorubrum trapanicum* DNA CBA1232	426	19948	0.15	7.03	46.8	+
NZ CP007055.1 *Halostagnicola larsenii* XH-48	1094	24861	0.39	8.91	22.7	
NZ CP008874.1 *Halanaeroarchaeum sulfurireducens* HSR2	596	15696	0.29	7.53	26.3	
NZ CP011564.1 *Halanaeroarchaeum sulfurireducens* M27-SA2	637	16067	0.30	7.55	25.2	
NZ CP016070.1 *Halodesulfurarchaeum formicicum* HTSR1	639	18453	0.32	9.36	28.9	
NZ CP016804.1 *Halodesulfurarchaeum formicicum* HSR6	696	19038	0.33	9.13	27.4	
NZ CP019067.1 *Halorientalis* sp. IM1011	1046	25987	0.31	7.68	24.8	+
NZ CP019285.1 *Halobiforma lacisalsi* AJ5	1235	40138	0.30	9.64	32.5	
NZ CP019327.1 *Haloterrigena daqingensis* JX313	1124	25935	0.33	7.63	23.1	
NZ CP019893.1 Natrialbaceae archaeon JW/NM-HA 15	1177	35113	0.30	8.93	29.8	+
Mean value per chromosome			0.42	6.34	18.4	
Standard Deviation			0.19	3.35	12.7	
NC 000913.3 *Escherichia coli* str K-12 substr MG1655	885	19124	0.19	4.12	21.6	+

$: Presence of a match to the *E. coli* DNA adenine methyltransferase in a translated nucleotide BLAST (tblastn) search with an *E*-value < 10^−25^ are indicated by +.

**Table 5:** DNA methylation patterns detected in *H. volcanii* RM deletion mutants

	*∆HVO_0794 ∆HVO_A0006 ∆HVO_A0237*		*ΔrmeRMS*		*ΔRM*	
Motif	GCA^m6^BNNNNNNVTGC	C^m4^TAG	GCA^m6^BNNNNNNVTGC	C^m4^TAG	GCA^m6^BNNNNNNVTGC	C^m4^TAG
Methylated position	3	1	3	1	3	1
Methylation type	6mA	4mC	6mA	4mC	6mA	4mC
Number of methylated motifs	410	0	0	1199	0	0
Number of motifs in genome	410	1342	410	1342	410	1342
Percent of methylated motifs	100	0	0	89	0	0
Mean modification QV score	213.0	-	-	104.1	-	-
Mean motif coverage	130.4	-	-	113.0	-	-

## Supplementary Materials

The following are available online at http://www.mdpi.com/2073-4425/9/3/129/s1. Figure S1. Growth curves of H26 and *ΔRM* when grown on Hv-YPC, represented by the average optical density (OD_620_) readings of 24 cell culture replicates taken each hour for 72 h of growth.
