# Supplementary material for: Characterizing the DNA Methyltransferases of Haloferax volcanii via Bioinformatics, Gene Deletion, and SMRT Sequencing
Source: Genes (Basel). 2018 Feb 27;9(3):129. doi: 10.3390/genes9030129 (PMC5867850; doi:10.3390/genes9030129)
Supplement: Supplementary file 1 [file genes-09-00129-s001.zip › Ouellette et al. 2018 Supplementary Figures/Ouellette et al. 2018 Supplementary Figure 1 Legend.docx]

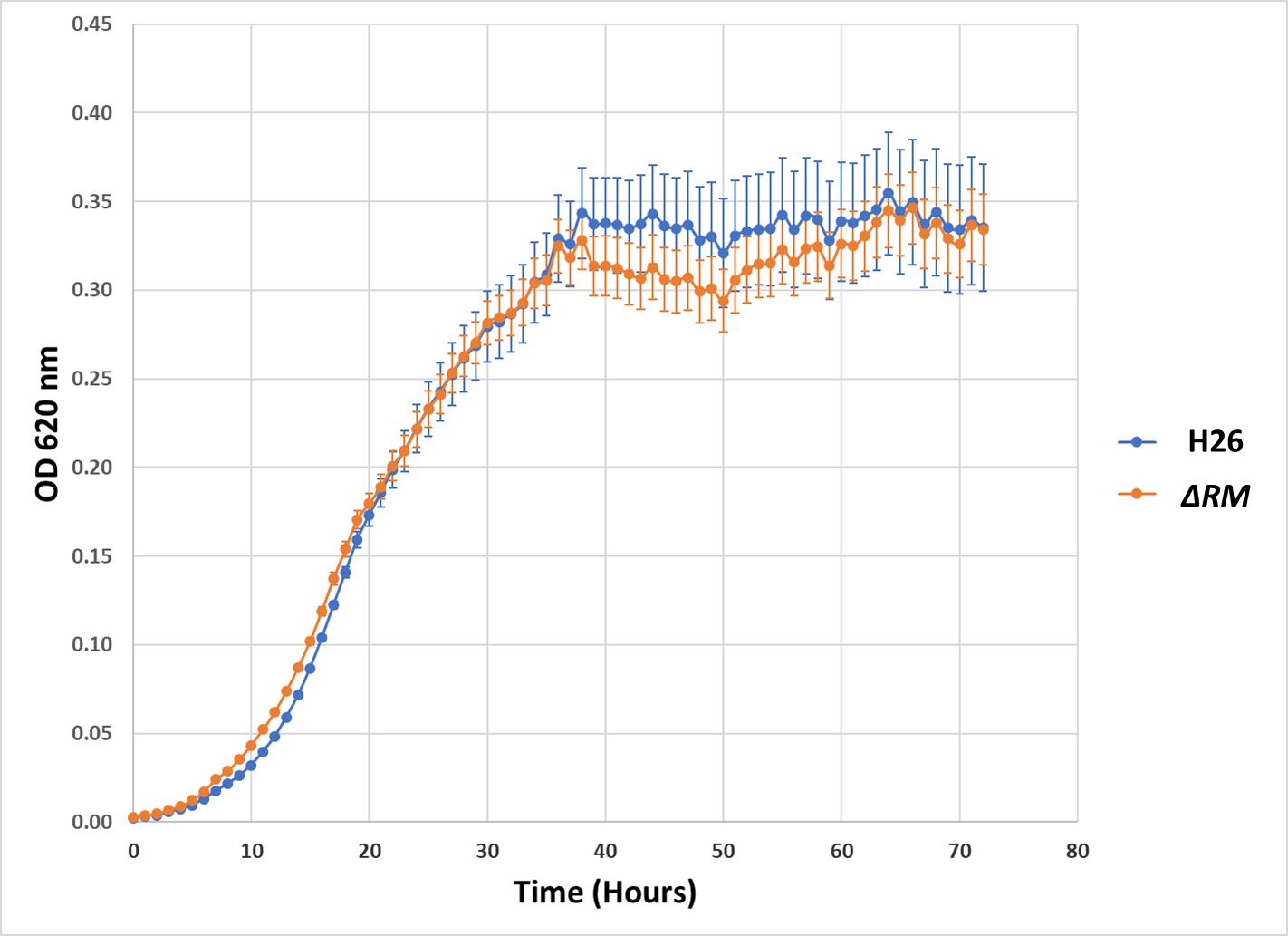


Supplementary Figure 1. Growth curves of H26 and ΔRM when grown on Hv-YPC, represented by the average optical density (OD_620_) readings of 24 cell culture replicates taken each hour for 72 hours of growth. Error bars indicate the standard error of the mean.
