# Supplementary figures and images for: Characterizing the DNA Methyltransferases of Haloferax volcanii via Bioinformatics, Gene Deletion, and SMRT Sequencing
Source: Genes (Basel). 2018 Feb 27;9(3):129. doi: 10.3390/genes9030129 (PMC5867850; doi:10.3390/genes9030129)

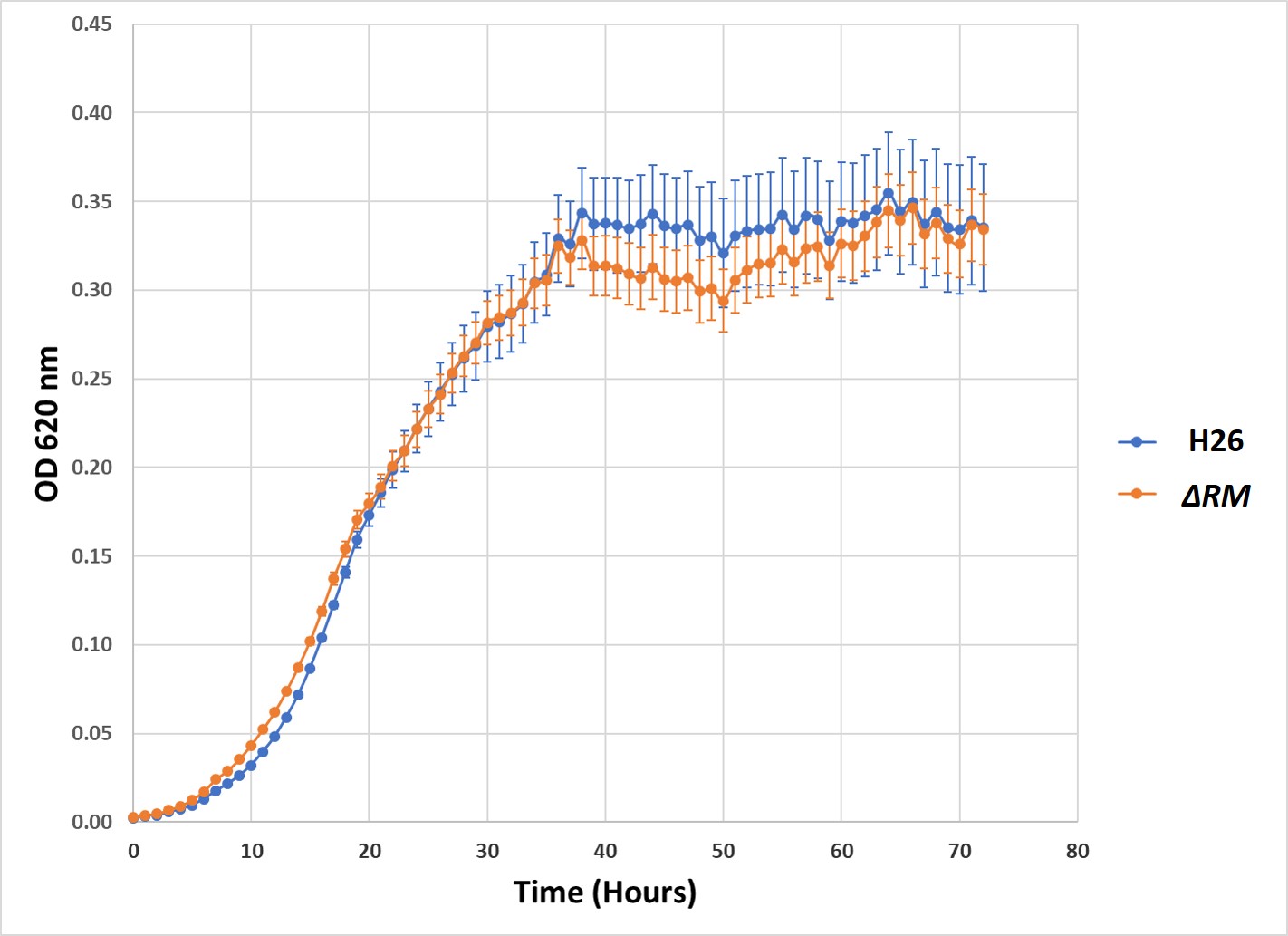

Supplement: Supplementary file 1 [file genes-09-00129-s001.zip › Ouellette et al. 2018 Supplementary Figures/Supplementary Figure 1.jpg]
